# Assessment of drug therapy problems and associated factors among PLWHA and tuberculosis admitted to two referral hospitals in Lusaka, Zambia

**DOI:** 10.1186/s12879-026-13024-z

**Published:** 2026-03-18

**Authors:** Divine Mbambala, Derick Munkombwe, Lungwani T. Muungu, Jimmy M. Hangoma, Martin Kampamba

**Affiliations:** 1 Department of Pharmacy, School of Health Sciences, University of Zambia, Lusaka, Zambia; 2 Department of Pharmacy, School of Health Sciences, Levy Mwanawasa Medical University, Lusaka, Zambia

**Keywords:** Drug therapy problems, PLWHA, Tuberculosis, Teaching hospital

## Abstract

**Background:**

Managing the simultaneous treatment of HIV/AIDS and tuberculosis (TB) increases the risk of adverse reactions, drug interactions, and challenges to treatment adherence. To address these complexities, a multidisciplinary approach is crucial for preventing, identifying, and resolving drug therapy problems (DTPs) in these patients. Therefore, this study aimed to assess the magnitude and associated factors of DTPs in hospitalized people with tuberculosis and those living with HIV/AIDs at two referral hospitals.

**Methods:**

We conducted a prospective observational study between January 2024 and May 2024 in the infectious diseases unit of the internal medicine department at the University Teaching Hospitals (UTHs) and Levy Mwanawasa University Teaching Hospital (LMUTH) . Cipolle’s and Strand’s DTP classification system was used to identify DTPs. Multiple logistic regression was used to evaluate the factors associated with drug-therapy problems.

**Results:**

A total of 139 patients were evaluated, of whom 117 (84.2%) presented with at least one drug therapy problem, with indication and effectiveness-relate problems being the most frequent.. The most common DTP was sub-therapeutic dosing, accounting for 92 (35.7%) cases. Of these, 74 (80%) were due to Dolutegravir sub-therapeutic dose, while 18 (20%) involved anti-tuberculosis medications. Poor adherence was the second most common DTP, occurring in 51 (19.8%) cases . The physician’s acceptance rate of clinical pharmacists’ interventions was 137 (98.1%). According to the multivariable, having tertiary education (AOR = 0.084, 95% CI: 0.011–0.66, *p* = 0.019), being divorced/widowed (AOR = 0.21,95% CI:0.056–0.79, *p* = 0.021) living outside Lusaka( Capital city) (AOR = 0.23,95% CI:0.054–0.995, *p* = 0.049) and self-employed(AOR = 0.02,95% CI:0.006–0.65, *p* = 0.021) were significantly associated with lower likelihood of with DRPs while being married (IRR = 1.58,95% CI:1.17–2.14, *p* = 0.003) and unemployed (IRR = 1.34,95% CI:1.04–1.76, *p* = 0.04) were associated with a higher incidence rate of DTPs.

**Conclusion:**

Drug therapy problems were highly prevalent among co-infected patients with HIV/AIDS and tuberculosis, with sub-therapeutic dosing and poor adherence being the most common. The high physician acceptance rate of clinical pharmacists’ interventions underscores the value of clinical pharmacy services in optimizing patient care. These findings highlight the need to strengthen clinical pharmacy services, particularly by increasing clinical pharmacists' involvement in multidisciplinary ward rounds to reduce drug therapy problems and optimize patient care.

## Introduction

The human immunodeficiency virus (HIV) and tuberculosis (TB) are two of the major infections that continue to be global health threats. HIV continues to infect about 1.5 million people each year and accounts for about 680,000 deaths from acquired immune deficiency syndrome (AIDS). Tuberculosis remains the leading single infectious cause of mortality worldwide, ranking second only to COVID-19 between 2019 and 2022, and causing approximately 1.2 million deaths annually [1, 2].

Evidence has shown that people living with human immunodeficiency virus/acquired immunodeficiency syndrome (PLWHA) are 26–31 times more likely to develop active tuberculosis (TB) compared to human immunodeficiency virus (HIV)-negative individuals due to the immunosuppressive effects of HIV on the host [3]. The prevalence of TB in HIV/AIDS varies across the world, with the highest prevalence (70%) reported in low- and mid-income countries. The prevalence of HIV/AIDS and TB coinfection in Zambia is increasing due to several risk factors, including poor economic conditions, substance abuse (e.g., alcohol, cocaine, etc.), low parental supervision, and peer pressure. Peer pressure has been shown to play a major role in tuberculosis infection as it shapes people’s social behaviours, such as spending more time in groups, attending crowded events or frequenting shared spaces (e.g., bars, clubs, social gatherings), which increase the risk of TB infections [4]. In 2021, an estimated 60,000 people were diagnosed with TB in Zambia (estimated incidence rate of 307 per 100,000 population per year), of whom approximately 50% were coinfected with HIV. This proportion is expected to increase [5].

According to Helper and Strand [6], drug therapy problems(DTPs) are any undesirable event related to medication therapy that has the potential to interfere with or actually interfere with the desired therapeutic outcomes. DTPs have been shown to affect treatment outcomes and increase the costs of therapy negatively. DTPs are categorized into seven major classes [7]. These include: unnecessary drug therapy, need for additional pharmacotherapy, sub-therapeutic dose, overdose, adverse drug reaction (ADR), need for a different medication, and non-compliance [8, 9]. Further, DTPs are broadly classified into four categories; indication, effectiveness, safety, and adherence. Drug therapy problems that affect effectiveness include ineffective drug therapy and sub-therapeutic dosing. Those affecting safety include adverse drug reactions and dosage too high. Finally adherence related DTPs include poor compliance [7]. If not attended to or adequately addressed, DTPs can result in harmful clinical outcomes ranging from temporary exacerbation of minor symptoms to permanent disability or death. In addition, the economic impact of DTPs is substantial and includes hospitalisations, litigation costs, hospital-acquired infections, lost income, and medical and disability expenses, costing some countries between US$6 billion and US$29 billion each year [10]. People with TB and HIV/AIDS are particularly susceptible to drug therapy problems due to several factors, including complex medication regimens, immunosuppression, stigma, adherence, comorbidities, and difficulties adhering to treatment guidelines. Both TB and HIV/AIDS require multiple medications with a high potential for drug-drug interactions and adverseeffects. Furthermore, the presence of other diseases due to immunosuppression also increases the likelihood of multiple drug use, thereby predisposing patients to polypharmacy. Identifying factors associated with the occurrence of DTPs in this population enables the healthcare team to implement targeted measures that aimed at preventing or reducing the magnitude thereby improve treatment outcomes. In a study conducted in the United States aimed at estimating the annual cost associated with prescription drug-related morbidity and mortality, the economic burden of non-optimised drug therapy was estimated to be $528.4 billion in 2016 US Dollars (USD) [2]. In this study, a non-optimised therapy was defined as any situation in which the prescribed medication regimen does not represent the most appropriate, effective, or safest therapy for the patient, potentially resulting in additional health complications, treatment failure, or death. Interventions by clinical pharmacists have been considered as a valuable contribution by the community in patient care by minimising medication errors, optimising medication therapy, and reducing the total cost of treatment [11].

Globally, several studies have highlighted the role of clinical pharmacists in identifying, resolving, and preventing DTPs across different hospital wards. However, in Africa, clinical pharmacy services remain undeveloped, and pharmacists largely perform traditional roles such as dispensing medications. A similar situation exists in Zambia, where pharmacists in most health facilities are primarily confined to pharmacy units and are rarely involved in clinical decision-making on hospital wards or in direct patient care alongside physicians. The limited integration of pharmacists into clinical care may increase the risk of drug therapy problems (DTPs).

Therefore, this study focused on exploring the magnitude of DTP and associated factors in PLWHA and TB at University Teaching Hospitals-Adult **(**UTHs) and Levy Mwanawasa University Teaching **(**LMUTH).

## Methodology

### Study design, period, and settings

A Hospital-based observational study was conducted between January 2024 to May 2024. Data were collected using a structured checklist from participants and their medical records, including patient files and ward prescription charts. The study was carried out at Levy Mwanawasa University Teaching Hospital (LMUTH) and the University Teaching Hospital’s adult Hospital (UTHs), both located in Lusaka, the capital city of Zambia. The UTHs is the largest tertiary care and teaching hospital in the country followed by LMUTH. The two hospitals serve a large population of approximately 3.32 million people residing in Lusaka, according to the 2023 population census [12].

### Study population and sample size determination

This study included both PLWHA and TB aged 18 years and above, who were receiving treatment for both conditions. Participants with incomplete medical records, those who did not provide consent to participate in the study, and patients receiving a modified anti-tuberculosis regimen secondary to liver and renal dysfunction were excluded.

The sample size was calculated using Cochrane`s formula: n =*Z*^2^*P (1-P)/d*^2^. Where n represent the required sample size, Z  is the confidence interval level (95%=1.96), and P is the   estimated population. An 80% prevalence of DTP was used in a main study from Brazil that evaluated the incidence of DTPs in PLWHA and TB patients [13]. n = (1.96)^2^(0.8) (1-0.8)/ (0.05)^2^*n* = 232. Based on the average number of patients admitted to the two hospitals over a four-month period, approximately 273 patients were expected to be available during the study period. Therefore, the adjusted sample size (nf) was calculated using the finite population correction formula:nf = (n × N)/(n + N), which yielded 125.4, and this was rounded up to 126 participants. Considering a potential non-response rate of 10%, the final minimum required sample size was 139 participants.

The probability proportional to size was used to allocate the sample size between the two Teaching Hospitals resulting in 105 participants from UTHs and 53 participants from LMUTH.

### Data collection tool

 DTPs were identified using the Cipolle and Strand classification system, a widely used patient-centred framework that provides a standardized guideline for clinical pharmacists in clinical practice while delivering pharmaceutical care [14]. According to this frame work, DTPs were classified into four main groups: indication, safety, efficacy and adherence [14]. Data collection and identification of DTPs were conducted by two trained Pharmacists, both holding Bachelor of Pharmacy degrees and fully registered with at least five years of practice and well-trained in the area of infectious diseases and antimicrobial resistance. Pharmacists obtained data directly from the patients and reviewed their medical records and prescription charts to identify potential and actual DTPs. Where appropriate, recommendations were made and any disputed cases were reviewed by a senior physician. The pharmacists were stationed at the two study sites, and received standardized training for data collection under the same ethical approval. The latest Zambian national treatment guidelines for HIV/AIDS and TB were used to assess the appropriateness of therapy and identify DTPs [15]. In addition, the pharmacists physically verified participants’ medications to ensure the appropriateness of the prescribed regimens taking into account clinical parameters such past medication history, drug allergies, comorbid conditions as well as laboratory parameters including viral load, CD4 cell counts, haemoglobin, serum creatinine, and liver function tests. Data were collected at a single point in time through patient interviews and review of medical records. The primary study outcome was the presence of drug DTPs, which was dichotomized as either present or absent. 

### Data analysis

Data were analysed using version 23 of the Statistical Package for the Social Sciences (SPSS). Initially, descriptive analysis was conducted to summarize the characteristics of the study population. This included frequency distributions for categorical variables and measures of central tendency for continuous variables, such as the mean. Measures of dispersion, including the standard deviation, were also calculated. In addition, descriptive analysis was used to determine the proportion of DTPs according to the proposed categories. The association between the selected variables (sociodemographic, behavioural, and clinical characteristics) and the occurrence of DTPs was assessed using logistic regression analysis. Variables with a p-value < 0.25 in the bivariate analysis were included in the multivariable logistic regression model. The Hosmer–Lemeshow goodness-of-fit test was performed to assess the adequacy of the logistic regression model. Variables with a p-value < 0.05 in the final model were considered statistically significant.

### Ethical considerations

Ethical approval was obtained from the University of Zambia Health Science Research Ethics Committee (UNZAHSREC), Protocol ID: 202,301,270,094. Permission to conduct the study and access to patient files and ward prescription charts was obtained from both the University and Levy Mwanawasa Teaching Hospitals’ management after ethical clearance and approval. The study involved direct contact with patients to obtain relevantinformation, which was subsequently verified through review of their medical records using a structures data collection sheet. Confidentiality was maintained throughout the study. Patient information and study results were securely stored and were not disclosed to individuals not involved in the research. Patient files and ward prescription charts were used as sources of data. To ensure anonymity, no personal identifiers were recorded; instead, unique serial numbers were assigned to each participant.

## Results

### Socio-demographic factors and clinical characteristics of PLWHA and TB admitted

A total of 139 PLWHA and tuberculosis were enrolled in the study (Table 1). The majority were male 75 (54.0%), aged above 40 years, 72 (51.8%), married, 66 (47.5%), and had primary education, 71 (51.1%). The mean age was 39.8 ± 12.1 years .

Most participants were unemployed 86, (61.9%), resided in Lusaka 117 (84.2%), and were living with family 130 (93.5%). Alcohol use and smoking were reported by 63 (45.3%) and 43 (30.9%) participants, respectively, while illicit drug use was reported by 54 (38.8%). The majority of participants had comorbidities 117 (84.2%), were in the first two months of tuberculosis treatment 128 (92.1%) and were taking ≥10 medications per day 114 (82.0%). Clinically, most participants were immunosuppressed with CD4 T-lymphocyte counts <200 cells/µL 92 (66.2%). Only 24 (17.3%) had an undetectable viral load (<20 copies/mL), while 42 (30.2%) had an unsuppressed viral load (>1000 copies/mL) (Tables 1 and 2)

**Table 1:** Sociodemographic and lifestyle characteristics of participants living with HIV/AIDS and tuberculosis (n = 139)

Variabl	Frequency (*n* = 139)	Percentage
**Gender**		
Male	75	54
Female	64	46
**Age**		
≤ 40 years	67	48.2
> 40 Years	72	51.8
**Marital Status**		
Married	66	47.5
Single	41	29.5
Divorced and widowed	32	23
**Level of Education**		
Non-Tertiary	133	95.7
Tertiary	6	4.3
**Employment Status**		
Employed	22	15.8
Unemployed	86	61.9
Self-employed	31	22.3
**Residential Address**		
Lusaka	117	84.2
Outside Lusaka	22	15.8
**Living Status**		
Living with family	130	93.5
Living with friends	9	6.5
**Cigarette Smoking**		
Yes	43	30.9
No	96	69.1
**Alcohol Consumption**		
Yes	63	45.3
No	76	54.7
**Illicit Drug Use**		
Yes	54	38.8
No	85	61.2
**Herbal Remedies**		
Yes	56	40.3
No	83	59.7

In many health studies, age 40 is often considered a threshold for increased risk of certain conditions such as hypertension, diabetes, cardiovascular disease, or chronic medication use). Categorising participants as < 40 and  ≥ 40 can therefore help highlight differences in health risks associated with ageing.

**Table 2:** Clinical characteristics of participants living with HIV/AIDS and tuberculosis (n = 139)

Variables	Frequency (*n* = 139)	Percentage
**Comorbidities Present**		
Yes	117	84.2
No	22	15.8
**Type of TB Diagnosis**		
Likely	98	70.5
Confirmed	41	29.5
**Time of TB Treatment**		
Within 2 months	128	92.1
Over 2 months	11	7.9
**Number of Drugs**		
< 10	25	18
≥ 10	114	82.0
**Viral Load Results**		
Not available	36	25.9
Undetectable (< 20 copies/mL)	24	17.3
Suppressed	37	26.6
Unsuppressed (> 1000 copies/mL)	42	30.2
**CD4** ^**+**^ **Count**		
Normal	21	15.1
CD4 T Lymphocyte < 200 cells/µL	92	66.2
Not Available	22	15.8

Comorbidities were categorised as non-communicable diseases (Hypertension, Diabetes mellitus, Cardiovascular diseases, Chronic kidney disease, Asthma / COPD, cancer) and communicable diseases (malaria, hepatitis B and opportunistic infections). This study assessed only the presence of comorbidities to explore their impact on DTPs and did not analyse the specific types of comorbid conditions. Among the participants, 21(15.1%) had no comorbidities, 17(12.2%) had communicable diseases comorbidities, and the majority 101(72.7) had non-communicable comorbidities, as shown in Table 3 below.

**Table 3:** Types of comorbidities

Type of comorbidity	*N* (%)
Participants without commodities	21(15.1)
Communicable diseases	17(12.2)
Non-communicable disease	101(72.7)

### Prevalence/frequency of drug therapy problems

**Fig. 1:**
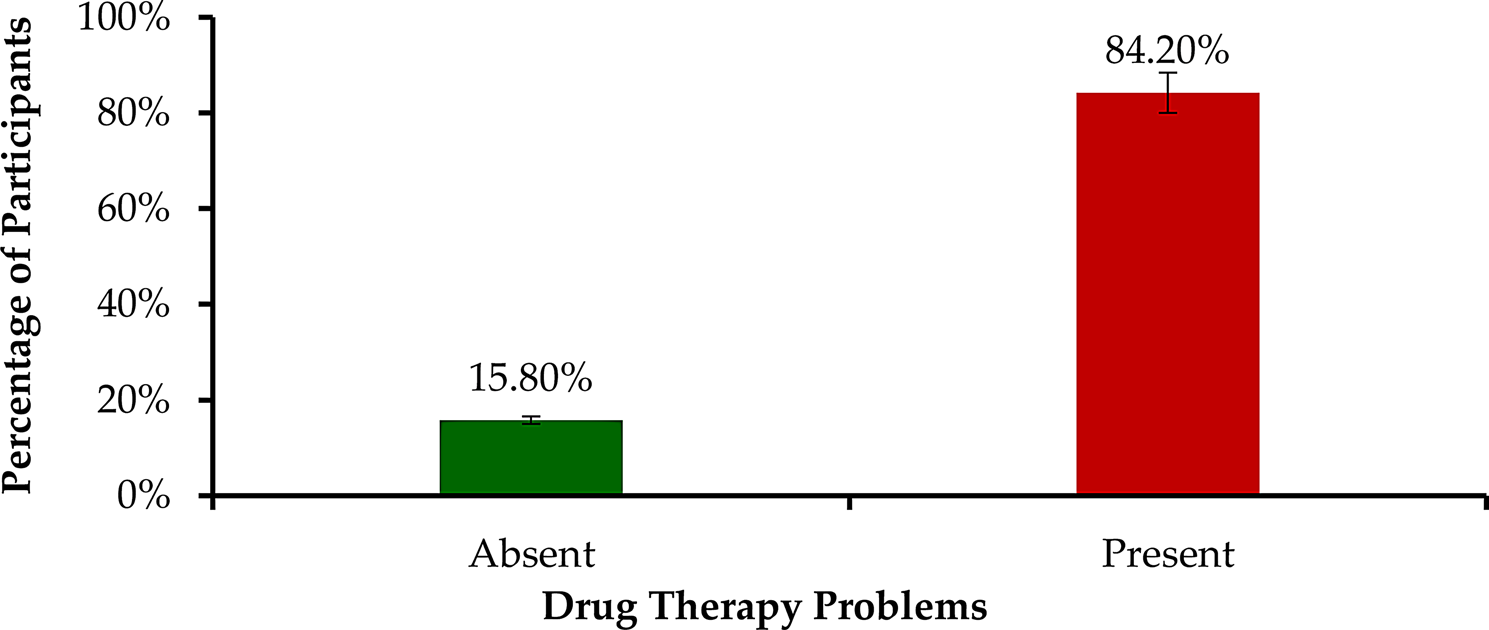
Prevalence of drug therapy problems (*n* = 139)

A total of 258 drug therapy problems (DTPs) were identified, with a mean (± SD) of 2.68 ± 1.7 DTPs per patient. Of the 139 participants assessed, the majority 117 (84.2%) had at least one DTP. (Fig. 1).

### The most frequently occurring drug therapy problem

Among the 258 identified drug therapy problems (DTPs), the most common was sub-therapeutic dosing 92 (35.7%). Of these, 207(80%) were attributed to sub-therapeutic dosing of dolutegravir and 57(20%) to anti-tuberculosis medications. The second most common DTP was poor adherence 51(19.8%). The main causes of poor adherence included patients’ preference not to take medication 155(60%), failure to understand instructions 78(30%), unavailability of medicines, and forgetting to take medications 26(10%). Additionally, 49 (19.0%) DTPs involved the need for a different medication. These cases included patients who continued receiving the same drug despite experiencing adverse reactions, as well as those receiving a less effective medicine when a more effective alternative was available (Table 4).

Table 4 shows that sub-therapeutic dosing was the most common DTP 92 (35.7%), followed by poor adherence 51(19.8%) and the need for a different medication 49(19%) (*n* = 258).

**Table 4:** The most frequently occurring DTP (n=258)

Type of drug therapy problEM	Frequency	Percentage
Adverse drug reaction	15	5.8
**Sub-therapeutic dose**	**92**	**35.7**
Needs additional pharmacotherapy	22	8.5
Unnecessary medication	13	5
**Poor adherence**	**51**	**19.8**
**Needs a different medication**	**49**	**19**
Overdose	16	6.2

### Hierarchical classification of drug therapy problems

**Fig. 2:**
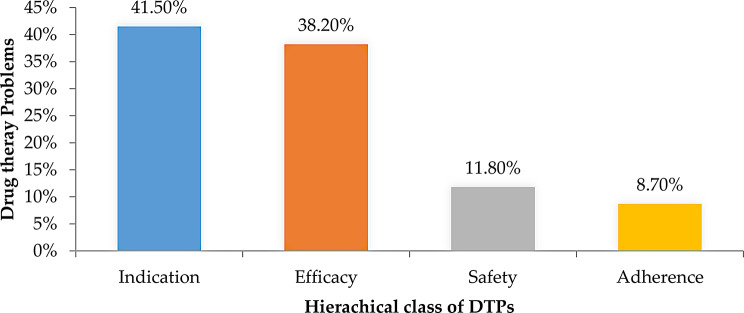
Hierarchical classification of DTPs

Based on the hierarchical classification of drug therapy problems (DTPs), most were related to indication 105 (41.3%), followed by effectiveness 97 (38.2%) (Fig. 2).

### The most implicated medications with drug-therapy problems

**Fig. 3:**
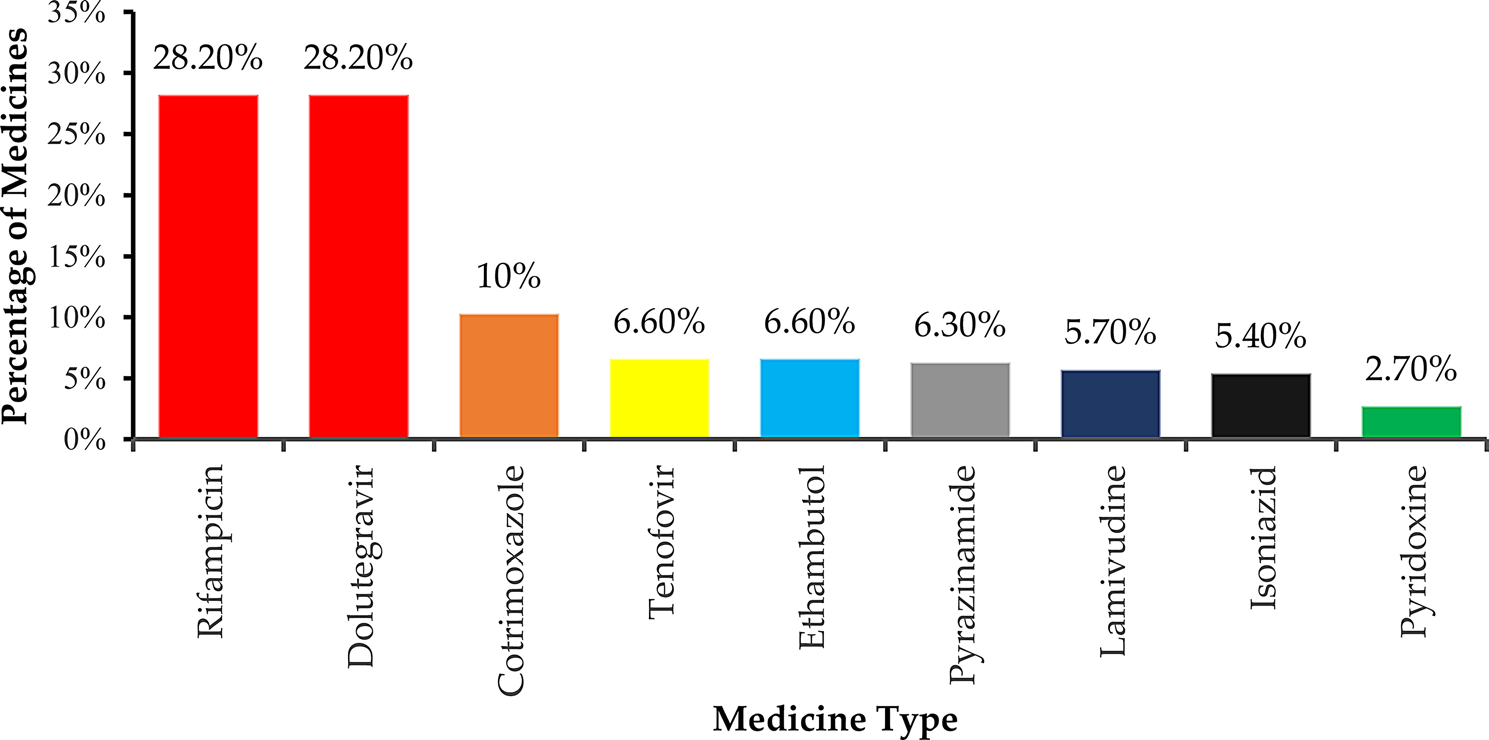
The most implicated medication

Rifampicin and dolutegravir were the medications most frequently implicated in drug therapy problems (DTPs), each accounting for 73 (28.3%), followed by co-trimoxazole 27 (10.5%), while pyridoxine was the least implicated, 7 (2.7%) (Fig. 3).

**Fig. 4:**
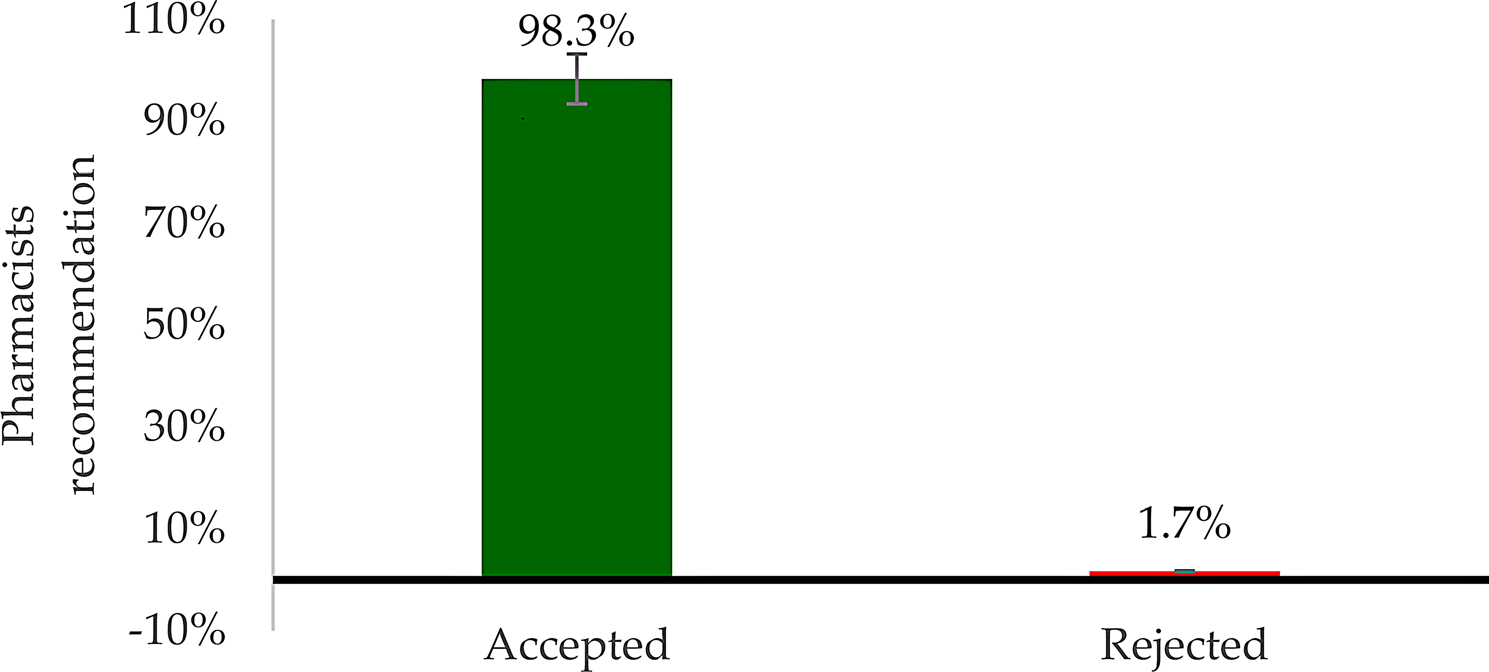
Physicians’ acceptance rate of clinical pharmacists’ recommendations

Out of 258 identified DTPs, 207(80.1%) required recommendations to physicians. These recommendations were mainly related to sub-therapeutic dosing, adverse drug reactions, the need for additional pharmacotherapy, and unnecessary medication. Of the 207 recommendations made, 203 were accepted by physicians.The acceptance rate of pharmacists’ recommendations in this study was 98.3%, while only 1.7% were rejected (Fig. 4). 

Table 5, shows interventions. Out of 139 participants, interventions were made in 116 participants; of these, 114(98.3%) were accepted, and 2(1.7%) were rejected.

**Table 5:** Intervention status (n=139)

Intervention status	*N* (%)
No intervention required	23(16.5%)
Intervention accepted	114(82%)
Intervention rejected	2(1.4%)

### Factors associated with drug therapy problems

The sociodemographic and clinical characteristics of people living with HIV/AIDS and tuberculosis with and without drug therapy problems (DTPs) were compared using bivariate logistic regression analysis. Patients aged above 40 years had a higher proportion of DTPs compared to those aged 40 years or below 72 (51.8%) vs. 67 (48.2%), p = 0.013. Participants residing outside Lusaka were less likely to experience DTPs compared to those residing in Lusaka 22 (15.8%) vs. 117 (84.2%), p = 0.031.Self-employed participants were less likely to have DTPs compared to those who were unemployed or formally employed 31 (22.3%) vs. 86 (61.9%) vs. 22 (15.8%), p = 0.025. Similarly, non-smokers were less likely to have DTPs than smokers 96 (69.1%) vs. 43 (30.9%), p = 0.028. Participants who did not use illicit drugs were less likely to experience DTPs compared to those who used illicit drugs 85 (61.2%) vs. 54 (38.8%), p = 0.014. In addition, participants who were not using herbal medicine were less likely to have DTPs than those who were using herbal medicine 83 (59.7%) vs. 56 (40.3%), p = 0.011 (Table 6).

**Table 6:** Factors associated with DTPs in hospitalized PLWHA and TB (*n* = 139)

Variables	Univariate analysis		Multivariable analysis	
	COR (95% CI)	*p*-value	AOR (95% CI)	*p*-value
**Gender**				
Male	Reference	0.95		
Female	1.03 (0.41–2.57)			
**Age**				
≤ 40 years	Reference	**0.013**		
> 40 Years	0.26 (0.09–0.75)			
**Marita Status**				
Married	Reference		Reference	
Single	3.46 (0.72–16.8)	0.12	0.847 (0.103-7.00)	**0.021**
Divorced and widowed	0.393 (0.144–1.07)	0.069	**0.21 (0.056–0.79)**	**0.021**
**Level of Education**				
Non-Tertiary	Reference	0.24	Reference	
Tertiary	**0.35 (0.06–2.1)**		**0.084 (0.01–0.66)**	**0.019**
**Employment Status**				
Employed	Reference		Reference	
Unemployed	0.36 (0.04-3.0)	0.35	0.55 (0.05–6.50)	0.631
Self-employed	**0.09 (0.01–0.73)**	**0.025**	**0.02 (0.01–0.65)**	**0.021**
**Residential Address**				
Lusaka	Reference	**0.031**	Reference	**0.049**
Outside Lusaka	0.32 (0.11–0.90)		**0.23 (0.054–0.995)**	
**Living Status**				
Living with family	Reference	0.69		
Living alone	1.48 (0.18–12.98)			
**Cigarette Smoking**				
No	0.19 (0.041–0.83)	**0.028**		
Yes	Reference			
**Alcohol Consumption**				
No	0.5 (0.14–1.1)	0.07		
Yes	Reference			
**Illicit Drug Use**				
No	**0.2 (0.06–0.73)**	**0.014**	**0.145 (0.02–0.72)**	**0.018**
Yes	Reference		Reference	
**Herbal Medicine use**				
No	0.19 (0.05–0.68)	**0.011**		
Yes	Reference			
**Comorbidity**				
No	0.82 (0.25–2.7)	0.74		
Yes	Reference			
**Mode of Diagnosis**				
Likely	Reference	0.2		
Confirmed	0.54 (0.21–1.40)			
**Time to Treating TB**				
Within 2 months	Reference	0.53		
Over 2 months	0.51 (0.06–4.20)			
**Number of Drugs**				
< 10	Reference	0.57		
≥ 10	0.68 (0.19–2.50)			

Variables with *P* < 0.25 in the bivariate analysiswere included in the multivariable logistic regression model. Alcohol use, smoking, and herbal medicine use were excluded from the final model due to multicollinearity (absolute correlation coefficient > 0.7) with illicit drug use. The overall model demonstrated a good fit, as indicated by a non-significant Hosmer–Lemeshow goodness-of-fit test (χ² = 12.9, df = 8, p = 0.116) and an R² value of 0.411. Multivariable analysis showed that having tertiary education (AOR = 0.084,95% CI:0.011–0.66, *p* = 0.019), not using illicit drugs (AOR = 0.145,95% CI:0.02–0.72, *p* = 0.018), being divorced and widowed (AOR = 0.21, 95% CI:0.056–0.79, *p* = 0.021), living outside Lusaka (AOR = 0.23,95% CI:0.054–0.995, *p* = 0.049) and self-employed (AOR = 0.02,95% CI:0.006–0.65, *p* = 0.021) were significantly associated with a lower likelihood of having DTPs. Results from the multivariable analysis model are shown in Table 7.

**Table 7:** The most frequently occurring DTP with specific examples (*n* = 258)

Type of drug therapy problem	*N* (%)	Examples
Adverse drug reaction	15(5.8)	Patients developed Hepatotoxicity, vision impairment, joint pain and Peripheral neuropathy from anti-TB (90%)
**Sub-therapeutic dose**	**92(35.7)**	Patients were receiving Dolutegravir 50 mg OD despite being on Rifampin (80%) and a sub-therapeutic dose of anti-TB medication.
Needs additional pharmacotherapy	22(8.5)	Immunosuppressed patients, but not prescribed Co-trimoxazole and, Pyridoxine not prescribed.
Unnecessary medication	13(%)	The patient with optic neuritis is still receiving ethambutol
**Poor adherence**	**51(19.8)**	Patients were not adhering to both ART and anti-TB medications.
**Needs a different medication**	**49(19)**	Patients with CKD yet on a TDF-based ARP regimen and those with eye issues but still on ethambutol
Overdose	16(6.2)	Overdose of Anti-TB and ART medication

Six (6) Variables were considered from the bivariate (p-value < 0.25) for poison multivariable analysis. According to the sensitivity test, being married (IRR = 1.58, 95% CI:1.17–2.14, *p* = 0.003) and unemployed (IRR = 1.34,95% CI:1.04–1.76, *p* = 0.04) were associated with a high incidence rate of DTPs. Details of the multivariable Poisson regression analysis are shown in Table 8.

**Table 8:** Multivariable poisson regression model predicting the total number of drug therapy problems as a secondary analysis

Variables	IRR	95% CI	*p*-value
**Marital Status**			
**Married**	**1.58**	**1.17–2.14**	**0.003**
Single	1.18	0.83–1.67	0.350
Divorced & widowed	Reference	Reference	
**Employment Status**			
Employed	1.13	0.77–1.64	0.541
**Unemployed**	**1.34**	**1.104–1.76**	**0.040**
Self-employed	Reference	Reference	

## Discussion

This study aimed to identify the prevalence and determinants of DTPs among hospitalised PLWHA and TB at two referral hospitals in Lusaka, Zambia. Previous studies have investigated DTPs or pharmacist-identified medication errors and related interventions among patients with tuberculosis [16] or HIV/AIDS [17] inseparately. However, evidence focusing on patients co-infected with both conditions remains limited, and heterogeneity in assessment methods hampers meaningful comparison across studies.

This study observed a higher prevalence of DTP 117(84.2%) compared with that reported in comparable primary studies [18, 19]. The high prevalence of DTPs may be attributed to limited clinical pharmacy services, a shortage of specialised pharmacists, and inadequate interprofessional collaboration. The mean of number (2.68) of DTPs per patient was comparable to findings from studies conducted in Ethiopia, possibly due to similarities in patient characteristics and study methodologies [20].

Sub-therapeutic dosing, poor adherence, and the need for different medication were the most common DTPs identified in this study. Sub-therapeutic dosing was mostly due to dolutegravir (80%), largely due to an unrecognised interaction between dolutegravir and rifampicin. Previous research suggests that sub-therapeutic dosing is a major contributor to treatment failure, particularly among PLWHA and TB [22]. According to the hierarchical classification of DTPs, indication-related probems were the most prevalent followed by efficacy and safety (41.3%, 38.9%, and 11.8% respectively). This finding is consistent with a study that was conducted in Brazil [13]. Dolutegravir and rifampicin were the drugs most commonly implicated in DTPs in the present study, in contrast to findings from a Brazilian study where pyridoxine was the most implicated drug.The acceptance rate of clinical pharmacists’ recommendations by physicians was high (98.3%) in the current study, which is consistent with findings from a study conducted in Spain among PLWHA. This finding in the present study highlights the value of integrating clinical pharmacists into patient care teams to improve medication management and enhance treatment outcomes [24].

Tertiary education was associated with a markedly lower risk of DTPs, with a 91.6% risk reduction compared to lower education levels. This finding is consistent with a study conducted in Turkey which reported that patients with low education attainment were twice as likely to experience DTPs compared to those with higher education [21]. Patients with tertiary education have better health literacy and are actively participate in healthcare decision-making [4]. In contrast, low educational attainment may limit patient's understanding of medications and treatment plans, thereby negatively influencing attitudes toward therapy and medication adherence [25]. 

This study found an association between DTPs and illicit drug use. Participants who did not use illicit drugs (AOR = 0.145, 95% CI: 0.02–0.72, *p* = 0.018) were less likely to experience DTPs. Similar studies among PLWHA and tuberculosis have not extensively explored this factor [13]. Substance use among PLWHA has been associated with psychological distress and reduced adherence to antiretroviral therapy [26]. Employment status was associated with DTPs, with self-employment linked to a lower likelihood of DTPs compared to formal employees (AOR = 0.02, 95% CI:0.01–0.65, *p* = 0.021). Previous studies have shown that self-employedindividuals may have greater flexibility and better work-life balance, which can facilitate adherence to medication schedules compared with those in full-time employment [27]. In addition, self-employed individuals may have improved access to health information and health insurance [28].

Regarding marital status, divorced and widowed participants (AOR = 0.21, 95% CI:0.056–0.79, *p* = 0.021) in the present study were less likely to experience DTPs compared to married participants. Some studies suggest that widowed or divorced individuals may develop adaptive coping strategies that enhance health awareness and medication adherence [29]. However, similar primary studies did not explore this factor among PLWHA and tuberculosis, highlighting an area for future research. Finally, participants living outside Lusaka (AOR = 0.23, 95% CI:0.054–0.995, *p* = 0.049) were less likely to experience DPTs compared to Lusaka residents. This finding is consistent with a study conducted in South Africa, which reported a higher prevalence of illicit drug use and depression among participants in urban areas. Depression has been associated with an increased risk of substance use and addiction, which may negatively affect health awareness and medication adherence.

The secondary analysis was conducted using the multivariable Poisson regression analysis to determine factors associated with the frequency (occurrence rate) of DTPs. The analysis revealed a significant association between marital status and the incidence rate of DTPs. Being married (IRR = 1.58, 95% CI:1.17–2.14, *p* = 0.003), was associated with a high incident rate of experiencing more DTPs than being widowed and divorced.Previous research suggests that married individuals may experience greater financial stress and increased responsibilities, particularly in urban settings, which may influence health behaviours and medication adherence [23]. In the current study, employment status was also associated with the incidence rate of DTPs. Unemployed participants were 1.34 times more likely to experience DTPs than those who were self-employed (IRR = 1.34, 95% CI:1.104–1.76, p-value = 0.04). The findings from the Poisson regression analysis were consistent with those obtained from the multivariable logistic regression model, particularly regarding the influence of marital status and employment status. These findings highlight the potential importance of socioeconomic factors in the occurrence of DTPs and may inform policymakers in developing strategies to improve economic stability and employment opportunities, which could indirectly contribute to better medication management and treatment outcomes. 

### Study strengths and limitations

This study has several strengths. Internal validity was strengthened through the use of multivariable logistic regression and Poisson regression analyses. In addition, the study was conducted at two major referral hospitals in Zambia, which enhances the generalisability of the findings to other referral hospitals with similar settings. The credibility of the findings is further supported by the fact that these two institutions serve a large population of approximately 3,324,000 people in Lusaka. Due to its cross-sectional design, causal relationships between identified factors and drug therapy problems (DTPs) could not be established. The study also did not assess the clinical outcomes or direct effects of DTPs on patients. Furthermore, as the study was conducted at public healthcare institutions, the findings may not fully represent the situation in private healthcare settings. Future studies using prospective cohort designs are recommended to better identify factors associated with DTPs in this population and to evaluate appropriate interventions.

The involvement of clinical pharmacists in tuberculosis and HIV care is essential for ensuring rational drug use, reducing medicine wastage, and improving medication adherence and treatment outcomes. These contributions enhance cost-effectiveness and resource efficiency, thereby supporting sustainable healthcare delivery, particularly in low-resource settings [30, 31]. 

### Implications of the findings

This study underscores a significant prevalence of DTPs among PLWHA and TB with sub-therapeutic dosing and poor adherence being the most common. The frequent occurrence of dolutegravir and anti-tuberculosis underdosing suggest inadequate attention to potential drug–drug interactions and weight-based dosing adjustments. The high acceptance rate of pharmacists’ interventions by physicians demonstrates the value of integrating clinical pharmacists into multidisciplinary healthcare teams. Furthermore, the occurrence of DTPs was significantly associated with educational level, employment status, and place of residence, indicating that socioeconomic factors and health literacy may influence medication use. These results highlight the need to strengthen clinical pharmacy services, enhance prescriber education, and promote patient-centred adherence support. In resource-limited settings, integrating pharmacists to routine HIV and TB care may improve therapeutic outcomes and optimise the use of healthcare resources.

## Conclusion

The study found that 84.2% of participants experienced drug therapy problems (DTPs), with a mean of 2.68 DTPs per patient. Sub-therapeutic dosing, poor adherence, and the need for a different medication were the most common types of DTPs. Factors significantly associated with DTPs included level of education, employment status, marital status, place of residence, and illicit drug use. The incidence rate of DTPs was higher among unemployed participants and lower among those who were divorced or widowed. These findings suggest that greater attention should be given to patients with these characteristics to reduce the occurrence of DTPs.

## Data Availability

The datasets used and/or analyzed during the current study are available from the corresponding author on reasonable request.

## Declarations

### Use of AI tools

The authors used Grammarly software for language editing purposes only. No generative AI tools (e.g., ChatGPT) were used in the preparation of this manuscript, and all intellectual content was developed by the authors.

## Abbreviations

DTP: Drug Therapy Problem
PLHIV: People Living with HIV
UTH: University Teaching Hospital
LMUTH: Levy Mwanawasa University Teaching Hospital

## References

[1] Olivier C, Luies L. WHO Goals and Beyond: Managing HIV/TB Co-infection in South Africa. SN Compr Clin Med. 2023;5(1):251.

[2] Watanabe JH, McInnis T, Hirsch JD. Cost of Prescription Drug-Related Morbidity and Mortality. Annals Pharmacotherapy. 2018;52(9):829–37. 10.1177/1060028018765159 29577766

[3] Herbert C, Luies L, Loots DT, Williams AA. The metabolic consequences of HIV/TB co-infection. BMC Infect Dis. 2023;23(1):536. 37592227 10.1186/s12879-023-08505-4 PMC10436461

[4] Marquez C, Chen Y, Atukunda M, Chamie G, Balzer LB, Kironde J, Ssemmondo E, Mwangwa F, Kabami J, Owaraganise A, et al. The Association Between Social Network Characteristics and Tuberculosis Infection Among Adults in 9 Rural Ugandan Communities. Clin Infect diseases: official publication Infect Dis Soc Am. 2023;76(3):e902–9. 10.1093/cid/ciac669 PMC10169405 35982635

[5] WHO. Global tuberculosis report. 2021.

[6] Niriayo YL, Kumela K, Kassa TD, Angamo MT. Drug therapy problems and contributing factors in the management of heart failure patients in Jimma University Specialised Hospital, Southwest Ethiopia. PLoS ONE. 2018;13(10):e0206120. 30352096 10.1371/journal.pone.0206120 PMC6198973

[7] Cipolle RJ, Strand LM, Morley PC. In: Pharmaceutical care practice: the patient-centred approach to medication management services, 3e. edn. New York, NY: The McGraw-Hill Companies; 2012.

[8] Strand LM, Cipolle RJ, Morley PC, Frakes MJ. The impact of pharmaceutical care practice on the practitioner and the patient in the ambulatory practice setting: twenty-five years of experience. Curr Pharm Design. 2004;10(31):3987–4001. 10.2174/1381612043382576 15579084

[9] PCNE. Pharmaceutical Care Network Europe: PCNE Classification. 2020, V5.01.

[10] Deawjaroen K, Sillabutra J, Poolsup N, Stewart D, Suksomboon N. Characteristics of drug-related problems and pharmacists’ interventions in hospitalized patients in Thailand: a prospective observational study. Sci Rep. 2022;12(1):17107. 36224350 10.1038/s41598-022-21515-7 PMC9556629

[11] Moges TA, Dagnew SB, Anberbr SS, Tarekegn GY, Yazie TS, Addis GT, Ayele TM, Setargew KH, Dagnew FN. Clinical pharmacists’ interventions about drug therapy problems and their acceptability by prescribers among pediatric hospitalised patients with infectious diseases in resource-limited settings. BMC Infect Dis. 2025;25(1):629. 40301792 10.1186/s12879-025-11044-9 PMC12042313

[12] CSO: Population and Demography of Zambia. Central Statistical Office; 2023.

[13] Resende NH, Miranda SS, Ceccato M, Haddad JPA, Reis AMM, Silva DID, Carvalho WDS. Drug therapy problems for patients with tuberculosis and HIV/AIDS at a reference hospital. Einstein (Sao Paulo Brazil). 2019;17(4):eAO4696. 31460617 10.31744/einstein_journal/2019AO4696 PMC6706227

[14] Cipolle RJ, Strand LM, Morley PC. Pharmaceutical Care Practice: The Patient-Centred Approach to Medication Management. Third Edition: McGraw-Hill LLC; 2012.

[15] Republic of Zambia MoH: Zambia consolidated guidelines for treatment and prevention of HIV infection. 2023, May 2022 version.

[16] Abrogoua DP, Kamenan BA, Ahui BJ, Doffou E. Pharmaceutical interventions in the management of tuberculosis in a pneumophtisiology department, Ivory Coast. Ther Clin Risk Manag. 2016;12:1749–56. 27920544 10.2147/TCRM.S118442 PMC5125718

[17] Aderemi-Williams RI, Nduaguba SO, Akoji EM, Ogbo PU, Abah IO. Drug therapy problems identified among patients receiving antiretroviral treatment in a HIV clinic: a prospective study in North Central, Nigeria. Pan Afr Med J. 2021;40:233. 35178144 10.11604/pamj.2021.40.233.28160 PMC8817191

[18] Detoni KB, Oliveira IV, Nascimento MM, Caux TR, Alves MR, Ramalho-de-Oliveira D. Impact of a medication therapy management service on the clinical status of patients with chronic obstructive pulmonary disease. Int J Clin Pharm. 2017;39(1):95–103. 27915426 10.1007/s11096-016-0402-6

[19] Dravid A, Natarajan K, Medisetty M, Gawali R, Mahajan U, Kulkarni M, Saraf C, Ghanekar C, Kore S, Rathod N, et al. Incidence of tuberculosis among HIV infected individuals on long-term antiretroviral therapy in private healthcare sector in Pune, Western India. BMC Infect Dis. 2019;19(1):714. 31409289 10.1186/s12879-019-4361-0 PMC6692924

[20] Bekele F, Fekadu G, Bekele K, Dugassa D, Sori J. Drug-related problems among patients with infectious disease admitted to medical wards of Wollega University Referral Hospital: Prospective observational study. SAGE Open Med. 2021;9:2050312121989625. 33552517 10.1177/2050312121989625 PMC7841694

[21] Kara E, İnkaya A, Aydın Haklı D, Demirkan K, Ünal S. Polypharmacy and drug-related problems among people living with HIV/AIDS: a single-centre experience. Turk J Med Sci. 2019;49(1):222–9. 30761883 10.3906/sag-1807-295 PMC7350848

[22] Ramachandran G, Chandrasekaran P, Gaikwad S, Agibothu Kupparam HK, Thiruvengadam K, Gupte N, Paradkar M, Dhanasekaran K, Sivaramakrishnan GN, Kagal A, et al. Subtherapeutic Rifampicin Concentration Is Associated With Unfavourable Tuberculosis Treatment Outcomes. Clin Infect diseases: official publication Infect Dis Soc Am. 2020;70(7):1463–70. 10.1093/cid/ciz380 PMC7931830 31075166

[23] Tang P, Pavlopoulou G, Kostyrka-Allchorne K, Phillips-Owen J, Sonuga-Barke E. Links between mental health problems and future thinking from the perspective of adolescents with experience of depression and anxiety: a qualitative study. Child Adolesc Psychiatry Mental Health. 2023;17(1):143. 10.1186/s13034-023-00679-8 PMC10740287 38129889

[24] Cantillana-Suárez MG, Robustillo-Cortés MLA, Gutiérrez-Pizarraya A, Morillo-Verdugo R. Impact and acceptance of pharmacist-led interventions during HIV care in a third-level hospital in Spain using the Capacity-Motivation-Opportunity pharmaceutical care model: the IRAFE study. Eur J Hosp pharmacy: Sci Pract. 2021;28(Suppl 2):e157–63. 10.1136/ejhpharm-2020-002330 PMC8640429 33627478

[25] Aje A, Segun S, Adisa R, Fakeye T, Olutayo A, Adebusoye L, Olowookere O. Effect of educational intervention on medication reconciliation practice of hospital pharmacists in a developing country – A non-randomised controlled trial. BMC Med Educ. 2023;23. 10.1186/s12909-023-04844-7 PMC10652589 37968602

[26] Sosengo T, Oridanigo E. Substance abuse and its association with adherence to ART drugs among HIV-positive pregnant women at selected hospitals of East Ethiopia. Sudan J Med Sci. 2023.

[27] Arpey NC, Gaglioti AH, Rosenbaum ME. How Socioeconomic Status Affects Patient Perceptions of Health Care: A Qualitative Study. J Prim Care Community Health. 2017;8(3):169–75. 28606031 10.1177/2150131917697439 PMC5932696

[28] Kvarnström K, Westerholm A, Airaksinen M, Liira H. Factors contributing to medication adherence in patients with a chronic condition: a scoping review of qualitative research. Pharmaceutics. 2021;13(7). 10.3390/pharmaceutics13071100 PMC8309154 34371791

[29] Onuh J, Mbah P, Ajaero C, Orjiakor T, Igboeli E, Ayogu K. Rural-urban appraisal of the prevalence and factors of depression status in South Africa. J Affect Disorders Rep. 2021;4:100082.

[30] Sajogo M, Teoh SWK, Lebedevs T. Pharmacist clinical interventions: Five years’ experience of an efficient, low-cost, and future-proofed tool. Res Social Administrative Pharm. 2023;19(3):541–6. 10.1016/j.sapharm.2022.12.008 36577571

[31] Lankford C, Dura J, Tran A, Lam SW, Naelitz B, Willner M, Geyer K. Effect of clinical pharmacist interventions on cost in an integrated health system speciality pharmacy. J Managed Care Speciality Pharm. 2021;27(3):379–84. 10.18553/jmcp.2021.27.3.379 PMC10391180 33645240

